# Scapular Free Flap for Soft Tissue Augmentation in Progressive Hemifacial Atrophy

**Published:** 2019-02-18

**Authors:** Ali Roham, Kongkrit Chaiyasate

**Affiliations:** Division of Plastic and Reconstructive Surgery, Beaumont Health System, Royal Oak, Mich

**Keywords:** progressive hemifacial atrophy, Parry-Romberg syndrome, scapular free flap, parascapular free flap, microvascular soft tissue augmentation

## DESCRIPTION

A 25-year-old female patient with a history of Parry-Romberg syndrome (PRS) and significant right facial soft tissue deficit presented to the reconstruction clinic for evaluation. She underwent reconstruction with a subscapular system free flap for facial augmentation utilizing a parachuting technique. After 2 revisions, she had excellent cosmetic outcome.

## QUESTIONS

1. What is the etiopathology of Parry-Romberg disease?
2. Which is the typical population affected by the disease?
3. What are the treatment options?
4. What is the anatomy of scapular free flap?

## DISCUSSION

Progressive hemifacial atrophy (PHA), also known as Parry-Romberg syndrome, was described as early as 1825 by Dr Caleb Parry.^1^ It is an infrequently encountered disease characterized by unilateral progressive skin, soft tissue, and muscle atrophy. This disease may involve the adjacent osteocartilaginous structure; however, this is primarily characterized as a hypoplastic state rather than an atrophic state.^2^ Although the etiology of the disease is not well known, Pensler et al^3^ described this as being a neurovasculitis along the branches of the trigeminal nerve. Other possible etiologies of the disease process include hereditary, autoimmune, traumatic, infectious, and alteration in the sympathetic nervous system.

PRS has a female predilection, with an onset in the first or second decade of life, progresses for 2 to 10 years, and then enters a stable phase. It has classically been described that the earlier the age of onset, less than 10 years, the more severe the facial deficit. Prior to the onset of disease, a cutaneous manifestation (eg, pigmentation change) has been described as well as cicatricial alopecia in the distribution of 1 or more branches of the trigeminal nerve.^4^

Treatment options for PRS include autologous lipofilling, pedicled flaps, alloplastic implants, and vascularized free tissue transfer.^5^ Nowadays, the most commonly used methods of soft tissue augmentation is by autologous lipofilling and microvascular free tissue transfer. The decision to proceed with autologous fat grafting versus free tissue transfer is based on severity of soft tissue deficit. Reconstruction with buried free flaps has been documented as having the lowest complication rates and lowest reoperation rates.^6^ Autologous fat grafting undergoes a 30% to 60% resorption of the transplanted fat and therefore may require repeat procedures.^7^

The scapular free flap has become the workhorse for facial augmentation in patients with PHA. The flap provides a long vascular pedicle with suitable diameter for microvascular transfer.^8^ There is minimal donor site morbidity with direct closure and no functional deficits postharvest. The subscapular system arises from the axillary artery and bifurcates to supply the scapular system via the circumflex scapular artery. This pedicle emerges from the tr iangular space, which is bounded by the teres major, teres minor, and long head of the triceps. Classically, the flap has been divided into the scapular flap and the parascapular flap, based on a transverse vascular branch and a longitudinal vascular branch, respectively. We describe a patient who required significant soft tissue augmentation utilizing a combined scapular/parascapular flap.

**Figure 1:**
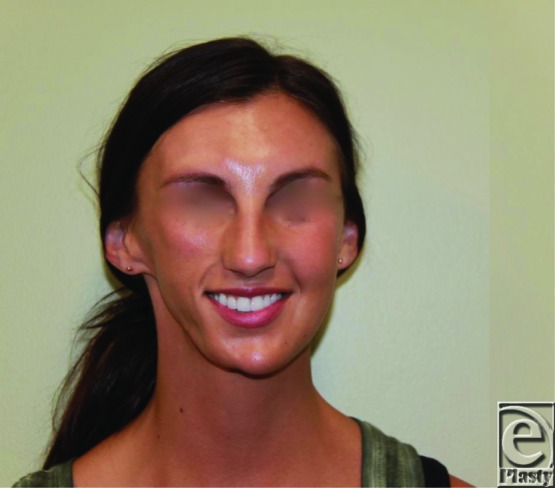
Patient preoperatively during her consultation showing significant right facial soft tissue deficit.

**Figure 2:**
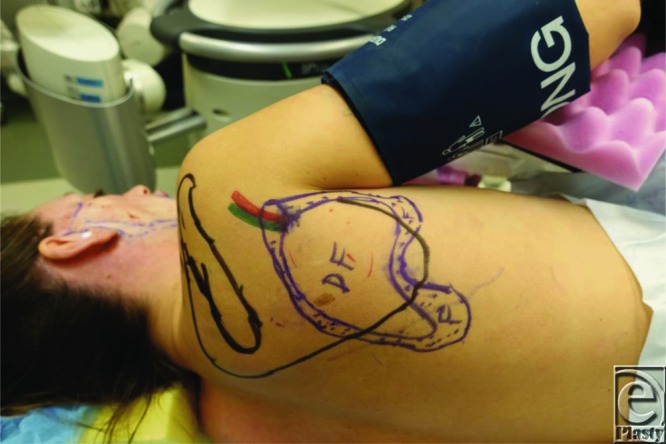
X-ray film used to transpose defect onto the scapular region.

**Figure 3:**
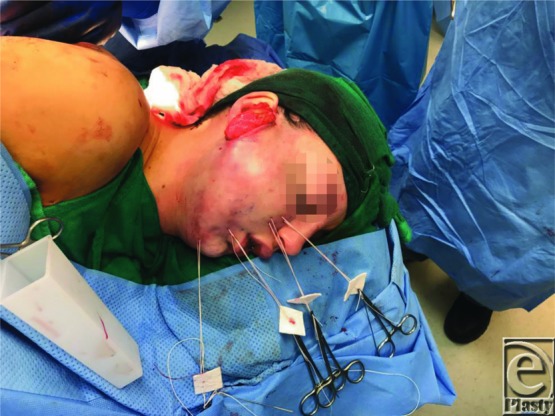
Following skin de-epithelialization, the flap was inset using transcutaneous sutures (parachuting technique).

**Figure 4:**
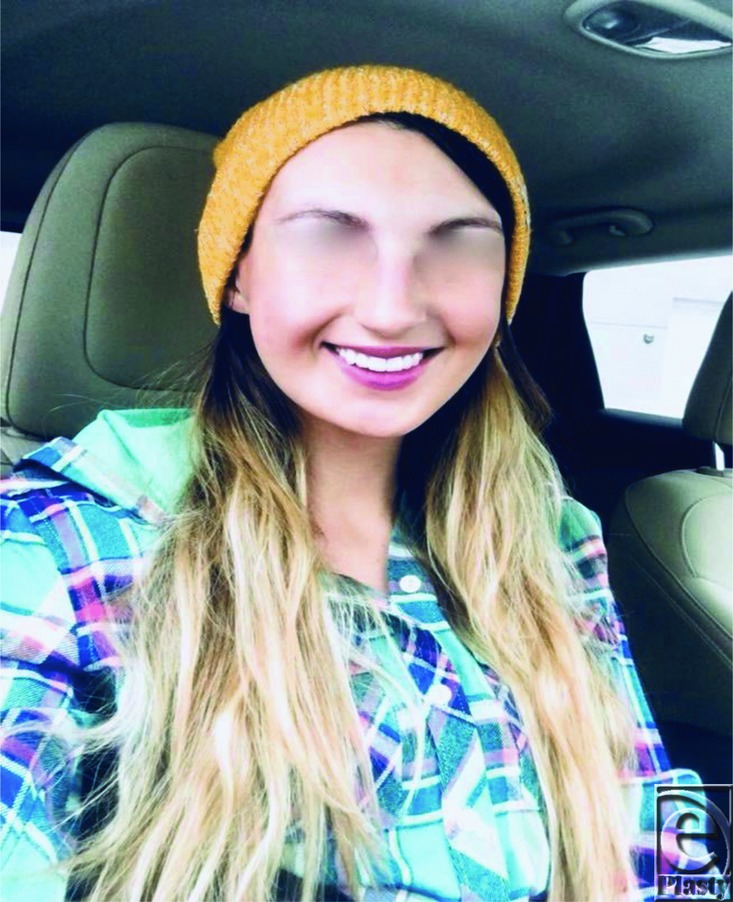
One year following initial surgery and 3 months following second revision. The patient with excellent cosmetic profile and facial animation.

A 25-year-old female patient with a history of PHA presented to our reconstructive clinic to discuss option for soft tissue augmentation. She stated her disease began in her early teens and had not noticed any additional change in several years. She did express the significant psychosocial effects the facial asymmetry had on her daily interactions with people. She had significant right facial soft tissue atrophy with preserved muscles of facial animation; therefore, we decided to proceed with free tissue transfer (Fig 1). The area of tissue deficit was outlined using a X-ray film and transferred to the scapular region for adequate tissue volume harvest (Fig 2). A scapular free flap was raised on the basis of the circumflex scapular system and anastomosed to the superficial temporal artery. The flap was appropriately oriented and utilizing a parachuting technique floated into its final position (Fig 3). She underwent 2 additional revisions with flap debulking and dermal fat grafting. At 1 year, she had an excellent aesthetic profile (Fig 4).
